# Vertical ozone formation mechanisms resulting from increased oxidation on the mountainside of Mount Tai, China

**DOI:** 10.1093/pnasnexus/pgae347

**Published:** 2024-08-22

**Authors:** Wanqi Wu, Yanzhen Ge, Yan Wang, Jixin Su, Xinfeng Wang, Bin Zhou, Jianmin Chen

**Affiliations:** Department of Environmental Science and Engineering, Shanghai Key Laboratory of Atmospheric Particle Pollution and Prevention (LAP3), Fudan University, Shanghai 200438, China; Tai’an Ecological Environment Protection and Control Center, Tai’an Ecological Environment Bureau, Tai’an 271000, China; School of Environmental Science and Engineering, Research Institute of Environment, Shandong University, Qingdao 266237, China; School of Environmental Science and Engineering, Research Institute of Environment, Shandong University, Qingdao 266237, China; School of Environmental Science and Engineering, Research Institute of Environment, Shandong University, Qingdao 266237, China; Department of Environmental Science and Engineering, Shanghai Key Laboratory of Atmospheric Particle Pollution and Prevention (LAP3), Fudan University, Shanghai 200438, China; Department of Environmental Science and Engineering, Shanghai Key Laboratory of Atmospheric Particle Pollution and Prevention (LAP3), Fudan University, Shanghai 200438, China

**Keywords:** ozone formation sensitivity regimes, vertical, nitrogen oxides, atmospheric oxidation capacity, mountainside

## Abstract

The vertical distribution of ozone (O_3_) within the boundary layer (BL) and its ground-level effects have been extensively studied. However, observational limitations in obtaining high-resolution, real-time data on O_3_ and its precursors, especially volatile organic compounds (VOCs), have led to a scarcity of research on O_3_ formation sensitivity and mechanisms. Online measurements for O_3_, nitrogen oxides (NO*_x_*), and VOCs were made on the mountainside of Mount Tai (∼550 m a.s.l.) in China during the summer of 2022 and were compared with the data from a ground-level site. The Master Chemical Mechanism (V3.3.1) was used to uncover a positive correlation between NO*_x_* and photochemical reaction rates on the mountainside, marking it as a NO*_x_*-limited regime in contrast to the VOC-limited regime identified at surface. On the mountainside, lower NO levels limited hydroxyl radicals (OH) recycling reactions, resulting in earlier O_3_ peaks and higher concentrations of hydroperoxy radicals (HO_2_) and organic peroxy radicals (RO_2_). The arrival of fresh air masses rich in NO accelerated OH radical cycling, enhanced atmospheric oxidization, and significantly impacted surface O_3_ concentrations though vertical transport. Moreover, NO*_x_* reduction scenario simulations show that when considering vertical transport, the peak O_3_ production rate at the surface is lower due to differences in O_3_ formation sensitivity vertically. This study highlights the significant sensitivity of O_3_ formation to NO within the BL, underscoring the potential impact of vertical in situ O_3_ formation above the ground on surface-level O_3_ concentrations through vertical exchange, particularly in cities with mountainous terrain.

**Significance Statement**

Observational limitations have hindered the acquisition of real-time, high-resolution data on O_3_ and its precursors, especially volatile organic compounds (VOCs), affecting in-depth research on vertical O_3_ formation. This study, covering a 3-month observation of O_3_ and its precursors at both mountainside and surface sites, reveals a significant sensitivity of low-altitude O_3_ formation to NO, particularly in cities surrounded by mountains, emphasizing the need for targeted attention. The results highlight the need for localized and refined emission reduction strategies that specifically address the variability in O_3_ formation sensitivity to NO at different atmospheric heights.

## Introduction

Ozone (O_3_), a key trace gas in the troposphere, has attracted sustained attention from scientific and regulatory communities over the past three decades due to its profound effects on human health, air quality, atmospheric oxidizing capacity (AOC), and climate (1, 2). Lower tropospheric O_3_ is mainly generated through photochemical reactions of its precursors, nitrogen oxides (NO*_x_*) and volatile organic compounds (VOCs). O_3_ concentrations are influenced by multiple factors, including background chemistry, regional chemistry, and local chemical production, as well as deposition and chemical removal (3). Planetary boundary layer (PBL) height, dynamics, and structure can affect O_3_ dispersion and transport (4, 5). As surface O_3_ pollution is closely linked with variations above the ground, it is essential to study the vertical O_3_ profiles for a thorough understanding of the region's O_3_ pollution within the PBL (6–8). Global Earth Observing System Chemistry (GEOS-Chem) simulations suggest that the primary reason for elevated O_3_ levels in southern China during summer is the circulation of O_3_ within the PBL (9). In eastern China, the significance of vertical transport is more pronounced in causing nocturnal O_3_ increase events than the concentrations of daytime O_3_ (10). Regional photochemically aged air masses from city clusters in the Yangtze River Delta substantially influenced early morning ground O_3_ concentrations by vertical mixing from the residual layer and contributed to boundary layer (BL) daytime O_3_ buildup (11). Research commonly focuses on the vertical transport of O_3_, but often overlooks the impact of in situ O_3_ formation within the PBL on ground-level O_3_ concentration.

Researching the O_3_ formation and sensitivity in the vertical direction is necessary. The low-altitude area just above the ground in the PBL is more affected by long-distance transport and has fewer emission sources, resulting in lower NO*_x_* levels. This could result in distinct mechanisms of O_3_ formation compared with the ground (12), particularly in urban areas with elevated NO_x_ concentrations. The expansion of the PBL and the turbulent mixing of fresh pollutants from the ground to the lower atmosphere complicate the study of O_3_ formation, emphasizing the need for in situ real-time monitoring of O_3_ and its precursors.

O_3_ formation sensitivity is usually classified into three regimes: VOC limited, transitional, and NO*_x_* limited. The determination of this sensitivity in the vertical direction is typically conducted through vertical profiles of characteristic pollutants, which are obtained via ground-based remote sensing. For example, the ratio of formaldehyde to NO_2_ (HCHO/NO_2_ or FNR) serves as a theoretical gauge of the relative abundance of total organic reactivity to hydroxyl radicals (OH) and NO*_x_*, and as such, it can function as a useful indicator of O_3_ sensitivity. Previous studies found that O_3_ formation sensitivity changed with altitude from VOC limited to transitional and NO*_x_* limited (13–17). However, changes in the HCHO/NO_2_ threshold in O_3_ regime classification modulated by meteorology and localized atmospheric chemistry in space and time, as well as uncertainties relating columns to the surface, preclude robust applications (18). While the observation-based model method alleviates some limitations, challenges persist in acquiring high-resolution model input parameters, such as detailed VOC data in vertical (19, 20).

Various methods are used to observe the vertical distributions of concentrations and compositions of VOCs, including mountaintop (21, 22), tall towers (23–26), tethered balloons (27–29), and aircraft measurements (30, 31). The Air Chemistry Research in Asia campaign utilized aircraft measurements over Hebei Province, China, to evaluate vertical profiles of O_3_, NO*_x_*, and VOCs alongside O_3_ production rates, determining that the O_3_ sensitivity regime across the PBL is NO*_x_* limited (32). However, offline sampling methods, such as tethered balloons and aircraft, result in a limited number of samples and large time intervals, which fail to accurately capture the true diurnal atmospheric variations. This limitation hinders the advancement of research into the in situ formation of O_3_ and the assessment of AOC (33–39). Mountain- and tower-based online VOCs measurements can provide detailed concentration levels and species composition. However, because mountain peak heights often exceed 1,000 m, they are typically considered as background sites. Additionally, high towers, affected by tourist activities, also cannot accurately represent the atmospheric composition and evolution in the region. Currently, there are very few studies that conduct online VOCs observation within the PBL and focus further on O_3_ formation sensitivity and mechanisms.

In recent years, the issue of O_3_ pollution in China has become increasingly prominent (40). Surface O_3_ observations reveal an increase rate of 1–3 ppbv per year over the megacity clusters in eastern China during 2013–2017 (41). The region around Mount (Mt.) Tai experiences severe O_3_ pollution, with mean concentrations of the 90th percentile of daily maximum 8-h O_3_ exceeding 180 μg/m^3^ in summer (42). Previous studies (3) have shown that high-altitude O_3_ in this region is highly sensitive to NO*_x_* in air masses from the southeast (account for 64% in July), and that surface emissions significantly impact background O_3_ concentrations. Therefore, investigating the sensitivity of O_3_ formation within the PBL and the relationship between local emissions and high-altitude O_3_ concentrations is essential for understanding the causes of O_3_ pollution in this region.

This study encompasses a 3-month observational period (June to August 2022) of O_3_ and its precursors at both the mountainside (∼550 m a.s.l.) and surface locations on Mt. Tai, in China. The pollution characteristics of O_3_ and its precursors at these two sites were examined. Utilizing an observation-based model, we focus on the in situ formation of O_3_ and its sensitivity to precursor compounds. In particular, this study provides insights into the accelerated radical cycling on the mountainside, which, in the presence of abundant NO, may result in the swift generation of O_3_, potentially influencing the surface. This work provides data support for studying the mechanisms of O_3_ formation within the PBL and reveals a significant sensitivity of low-altitude O_3_ formation to NO, particularly in mountainous regions. It also offers insights into exploring the causes of ground-level O_3_ pollution and developing further control strategies.

## Results

### Overview of O_3_ and its precursors at both the mountainside and surface sites

**Fig. 1.**
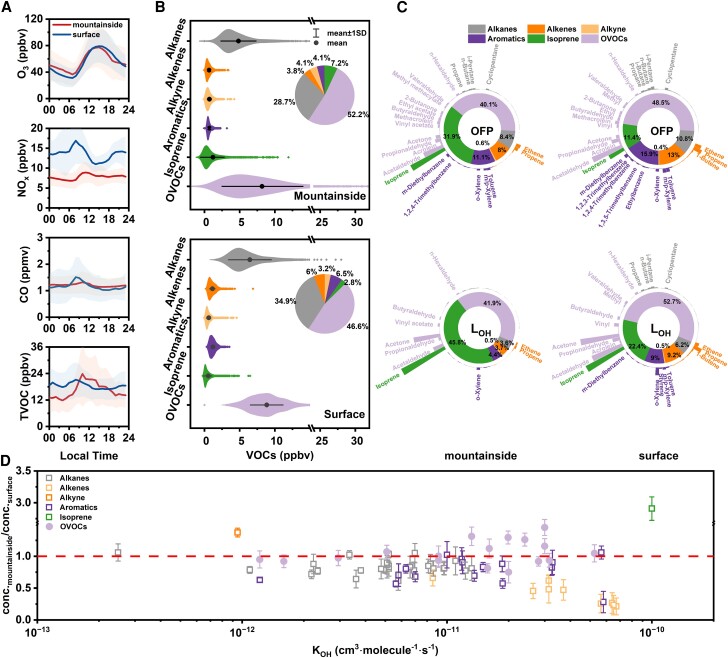
A) Diurnal patterns of O_3_, CO, NO_2_, and TVOCs. Lines represent the average values, and shaded areas represent the SDs. B) Concentration and proportional composition of VOC groups at the two sites. C) Proportions of VOC groups (inner ring) and VOC species (outer bar) according to the OFP and L_OH_ at the two sites. The top VOC species that contributed >2% are marked on the outer bar. D) Daytime relationship between the mountainside-to-ground ratio of VOC species and their rate coefficients for reaction with OH.

The diurnal patterns of O_3_ and its precursors are shown in Fig. 1A. The peak concentration of O_3_ was similar for the mountainside and the surface, whereas the mountainside site experienced the peak earlier than the surface site, attributed to limited NO*_x_* (as discussed in the Diverse photochemistry and its sensitivity to O_3_ precursors section). Furthermore, the mean nighttime and early morning concentrations on the mountainside exceeded those at the surface owing to reduced NO titration. The NO*_x_* level observed on the mountainside (7.8 ± 2.1 ppbv) was significantly lower than that measured at the surface (13.6 ± 3.7 ppbv). Surface NO*_x_* concentrations peaked during morning and afternoon rush hours, followed by a delayed increase on the mountainside. Such patterns, also noted for CO and VOCs, are attributed to the vertical air mass exchange driven by BL expansion. Figure S1 shows the diurnal variations of some VOC species, also revealing the phenomenon of delayed peaks on the mountainside. Moreover, the NO*_x_* concentration on the mountainside was consistently lower than at the surface site, exhibiting a 26.7% reduction during midday minimums and a 58.2% reduction during traffic peaks. This pattern suggests an average vertical gradient of 5.8 ppbv between the two sites, narrowing to ∼2.9 ppbv by late afternoon, likely due to enhanced photolytic NO_2_ loss efficiency at the surface. The diurnal variation of CO concentrations on the mountainside remained relatively stable, with fluctuations not exceeding 13.2%, in stark contrast to the surface, where variations reached up to 32.3%. The concentrations of measured total VOCs (TVOC) on the mountainside (16.8 ± 8.3 ppbv) were lower than those on the surface (18.5 ± 6.1 ppbv). However, during the daytime hours, the concentrations on the mountainside exceeded those at the surface, primarily due to the abundant plant sources on the mountainside, which emitted large amounts of isoprene.

The compositions of the VOC groups at the two sites are compared in Fig. 1B. Notably, the contribution of isoprene, driven by biological emissions, was higher on the mountainside (7.2%) compared with ground level (2.8%), indicating a potentially significant impact on photochemical mechanisms. Oxygenated VOCs (OVOCs) were the most abundant VOC category at both the mountainside and ground level, with their average concentrations measured at 8.8 ± 5.8 and 8.6 ± 2.5 ppbv, respectively. The elevated proportion of OVOCs, accounting for 52.2% of the TVOC on the mountainside compared with 46.6% at the ground level, suggests a greater degree of air mass aging at higher elevations (as discussed in the Potential sources of VOCs section). Alkanes, alkenes, acetylene, and aromatics constituted 28.7, 3.8, 4.1, and 4.1% of the TVOC on the mountainside, respectively, compared with 34.9, 6, 3.2, and 6.5% at the surface, respectively. This distribution underscores the varied VOC profiles between the two sites, as detailed in Tables S1 and S2.

Figure 1D shows the daytime relationships between the ratios of VOC species concentrations from the mountainside to the surface and their reaction rate coefficients with OH radicals. Most species, except for some OVOCs and aromatic compounds displayed ratios below 1, indicating lower concentrations on the mountainside compared with the surface. Moreover, the reduction in the ratios of most VOC species is related to increased reaction rates with OH radicals, especially in alkenes. This indicates that as the air masses travel from fresh emission sources to the mountainside, reactive VOCs continuously react and decrease. Conversely, OVOCs, mainly formed secondarily, exhibited a different trend. Interestingly, the ratio of some reactive aromatic compounds was above 1, implying additional aromatic emissions on the mountainside. Additionally, the daytime isoprene concentrations on the mountainside were significantly higher than at the surface, with a ratio (marked by a green box) of 2.9, highlighting the critical role of isoprene in photochemical oxidation at the mountainside (as discussed in the Diverse photochemistry and its sensitivity to O_3_ precursors section).

The atmospheric reactivity of VOCs reflects each VOC's ability to participate in chemical reactions. The ozone formation potential (OFP) (43) and OH radical loss rate (*L*_OH_) (44), which determine the O_3_ formation capability of VOCs, were calculated. The results are presented in Fig. 1C and Table S3; references for methods are available in the Supplementary material. The OFP values were 119.1 ± 83.0 μg/m^3^ on the mountainside and 147.5 ± 46.7 μg/m^3^ at the surface. OVOCs were the predominant contributors to the OFP at both sites, accounting for 40.1 and 48.5% of the total OFP, respectively. Key contributors included isoprene, acetaldehyde, propionaldehyde, acetone, ethene, and propene. The *L*_OH_ value ranged from 0.03 to 3.02 s⁻^1^ across different VOC groups, with cumulative *L*_OH_s of 6.3 ± 5.8 s⁻^1^ on the mountainside and 5.7 ± 1.8 s⁻^1^ at the surface, the former being markedly higher due to elevated isoprene levels. Given the limited reports on OH radical loss rates within the PBL, the mountainside's *L*_OH_ value was compared with summer ground-level reports from major Chinese cities. The *L*_OH_ on the mountainside was lower than Beijing (8.3 s⁻^1^) (34), closely aligned with Zhengzhou (6.7 s⁻^1^) (36), and significantly higher than Shanghai (3.2 s⁻^1^) (35). These comparisons suggest that photochemical reactions are more intense on the mountainside, indicating the need for an in-depth analysis of the sources of O_3_ precursors there to determine the causes for these strong photochemical reactions.

### Potential sources of VOCs

**Fig. 2.**
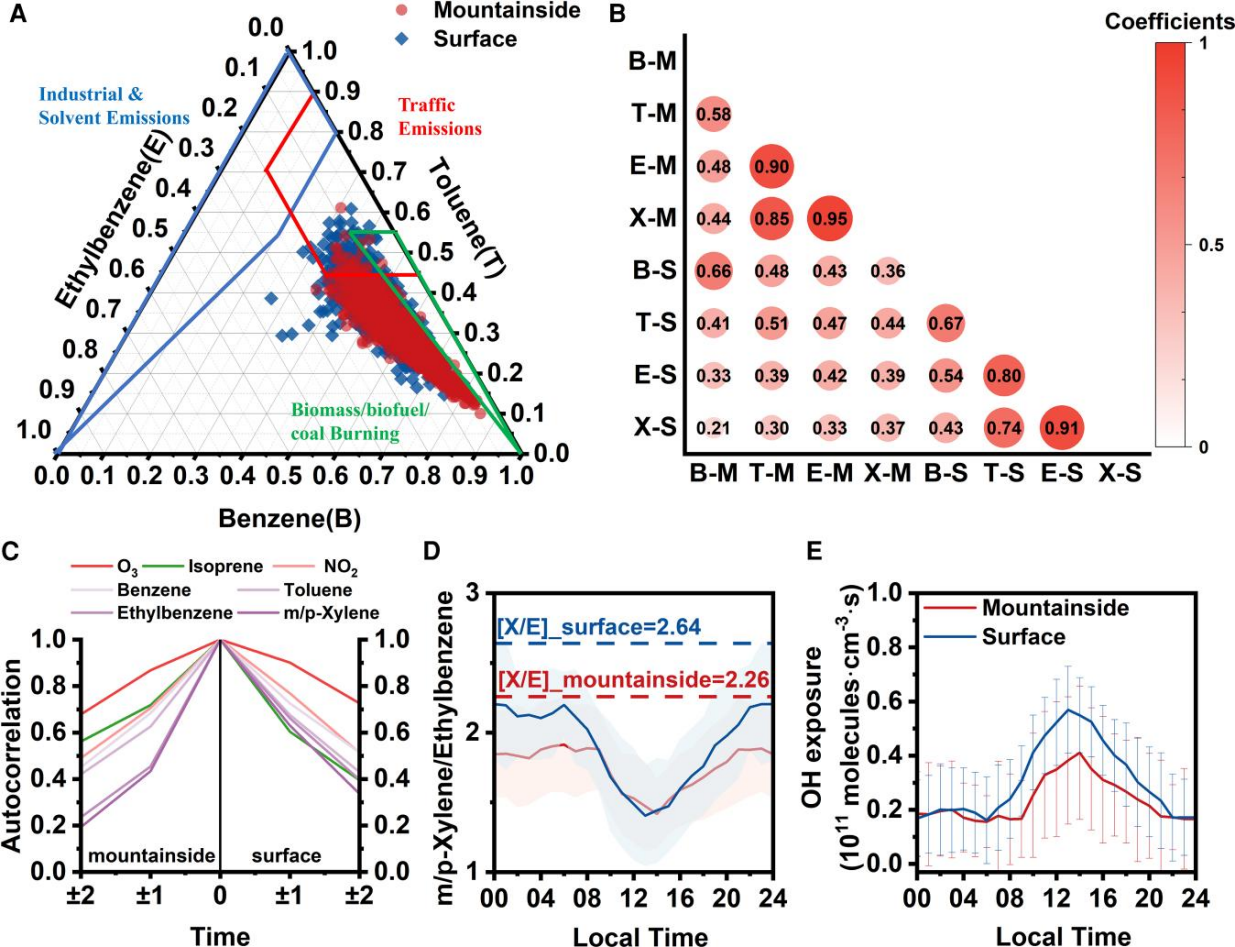
A) Relative proportions of benzene (*B*), toluene (*T*), and ethylbenzene (*E*) at the two sites. B) The correlations of *B*, *T*, *E*, and *m*/*p*-xylene (*X*) are illustrated for the mountainside (M) and surface (S) samples. The size and color of the circles represent the relative strength of the correlations, and the numbers indicate the specific values. C) Autocorrelation of the time series of O_3_, NO_2_, and selected VOC species. D) Diurnal variation in *m/p*-xylene to ethylbenzene ratio. E) Calculated OH exposures based on observations.

To further explore the potential sources of VOCs at both sites, we investigated the mean diurnal variations in tracer VOCs, as shown in Fig. S1. Elevated afternoon isoprene levels indicated significant biogenic emissions. Serving as tracers for motor vehicle emissions (45), *n*-pentane and *i*-pentane exhibited notable peaks at 08:00 local time (LT), reflecting vehicular activity. Additionally, benzene (*B*), toluene (*T*), ethylbenzene (*E*), and *m*,*p*-xylene (*X*) displayed similar diurnal patterns, underscoring the considerable influence of motor vehicles on VOC emissions. The toluene-to-benzene (*T*/*B*) ratio, used for source identification, offers distinctive value for categorizing specific sources, such as vehicle emissions (2.0), industrial processes (3.0–6.9), fuel evaporation (4.1), coal and biomass burning (0.2–0.6), and the use of paint solvents (11.5) (3). The *T*/*B* ratio averaged 0.35 (range: 0.12–2.01) on the mountainside and 0.71 (range: 0.19–1.92) at the surface, highlighting significant contributions from biomass burning and vehicle emissions at both sites. To refine source discrimination amid overlapping *T*/*B* ratios, a ternary diagram (46) was employed. The ternary fractions of benzene:toluene:ethylbenzene (*B*:*T*:*E*) averaged 0.59:0.31:0.11 on the mountainside and 0.52:0.37:0.11 at the surface, respectively. Samples scattered across the ternary plot (Fig. 2A) indicate a significant impact from mixed emission sources, particularly from traffic and biomass/biofuel/coal burning, during the sampling period.

VOCs at both the mountainside and ground level exhibit similar emission sources. Moreover, the observed delay in peak concentrations of tracer VOCs, CO, and NO*_x_* on the mountainside (as shown in Fig. 1A and Fig. S1), coupled with the diminishing ratios of VOCs between the mountainside and the ground level as a function of their OH radical reaction rates (Fig. 1D), collectively underscore the dynamics of pollutant dispersion across the vertical gradient. Figure 2B shows the strong positive correlations between key tracer VOCs at both sites, indicating the significant influence of ground-level O_3_ precursors on both the concentrations of these precursors and O_3_ formation processes on the mountainside. To further explore the spatial extent of emission sources for VOC species on the mountainside, autocorrelation profiles of their time series were calculated by offsetting the time from −2 to 2 h, as shown in Fig. 2C. As found in previous studies (25), the concentration profiles of species mainly affected by local sources tend to have narrower autocorrelation profiles. The differences in autocorrelation profile widths for various species on the mountainside indicate that these species come from a mix of sources across larger areas. Specifically, the autocorrelation profile for isoprene on the mountainside was narrower (even more so than for NO_2_), suggesting a close link to local mountain emissions.

This phenomenon, indicating the prevalence of more aged air masses on the mountainside, is further supported by the lower initial mixing ratio of *m*/*p*-xylene to ethylbenzene (Fig. 2D). The reactivity of *m*/*p*-xylene with OH radicals was 2.7 times greater than that of ethylbenzene (43); therefore, a lower *m*/*p*-xylene/ethylbenzene (*X*/*E*) ratio represents a greater degree of air mass aging (47). Thus, the initial mixing ratio of *X*/*E* on the mountainside was 2.26, compared with 2.64 at the surface, demonstrating more aged air masses on the mountainside. A photochemical age-based parameterization method for OH exposure, detailed in the Supplementary material, was used to calculate the photochemical aging of air masses from emissions to observations, with lower OH exposure, indicating more aged air masses (47–49). The diurnal variations in OH exposure exhibited an inverse correlation with the *X*/*E* ratio, peaking daily between 12:00 and 15:00 (Fig. 2E). The average OH exposure on the mountainside was measured at 2.3 × 10^10^ molecules cm^−3^ s, notably lower compared with the surface value of 3.0 × 10^10^ molecules cm^−3^ s. Compared with previous studies, the OH exposures were within the range observed at two urban sites in Beijing (6.3 × 10^10^ molecules cm^−3^ s) (47) and Xi’an (1.7 × 10^10^ molecules cm^−3^ s) (50), and close to that in Los Angeles (3 × 10^10^ molecules cm^−3^ s) (51). It is noteworthy that the SD of OH exposure on the mountainside was greater than at the ground level. This indicates that significantly different environmental conditions on different days affected the mountainside values, thereby influencing the reactions between VOCs and OH radicals on the mountainside. Since OH radicals are the most important oxidizing agents in the atmosphere, substantial differences in photochemical reactions must exist between the two sites.

### Diverse photochemistry and its sensitivity to O_3_ precursors

**Fig. 3.**
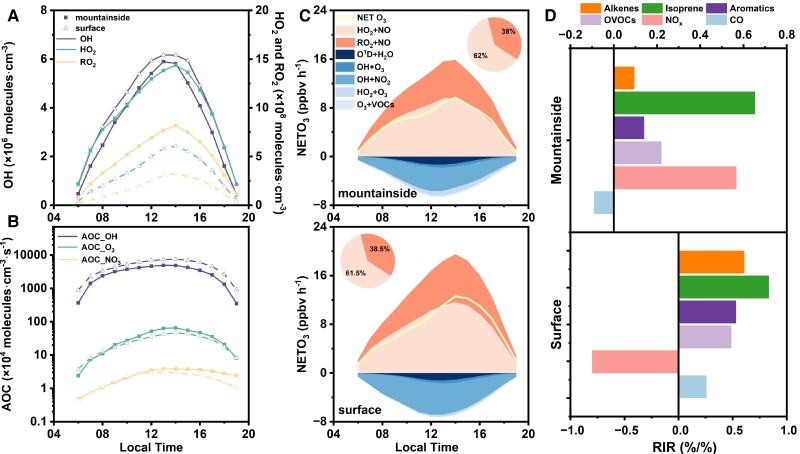
A) Daytime variations in the concentrations of OH, HO_2_, and RO_2_ radicals. B) Percentages of OH, O_3_, and NO_3_ in the AOC. C) The hourly variation in O_3_ production and destruction rates at both sites. Pie charts represent the portions of different reaction channels. The yellow lines represent the net O_3_ generation rate. D) RIR of the VOC groups, NO*_x_* and CO for O_3_ during high O_3_ daytime (06:00–18:00 LT).

The distinct VOCs profiles and lower NO*_x_* levels on the mountainside reveal significant differences in photochemical reactivity, underscoring the critical roles that VOCs and NO*_x_* play as primary O_3_ precursors. We used the Master Chemical Mechanism (MCM) (V3.3.1) to simulate the concentrations of OH radicals, hydroperoxy radicals (HO_2_), and organic peroxy radicals (RO_2_), along with their photochemical reaction rates. Figure 3A shows the daytime average simulated concentrations of these radicals. The average OH radical concentrations on the mountainside were (3.3 ± 2.3) × 10^6^ molecules cm^−3^, slightly lower than those at the surface [(3.9 ± 2.1) × 10^6^ molecules cm^−3^]. Notably, the OH radicals peaked earlier on the mountainside, paralleling the daily O_3_ pattern. On days with high O_3_ levels, as shown in Fig. S2, peak arrival times were delayed, indicating an increased presence of NO, which facilitates reactions with HO_2_ and RO_2_ radicals. As shown in Fig. 3A, the mountainside exhibited higher concentrations of HO_2_ and RO_2_ radicals, where limited NO availability constrained OH recovery at noon. A similar phenomenon was observed in a comparison of 10 rural and urban sites in eastern China (52). On the mountainside, the HO_2_ and RO_2_ radical concentrations were (9.2 ± 6.4) × 10⁸ and (4.8 ± 3.9) × 10⁸ molecules cm^−3^, respectively, in contrast to (3.2 ± 2.3) × 10⁸ and (1.7 ± 1.3) × 10⁸ molecules cm^−3^ at the ground level.

The AOC is calculated as the sum of the oxidation rates of various primary pollutants (CO, NO*_x_*, VOCs, etc.) by the major oxidants (i.e. OH, O_3_, and NO_3_), as detailed in the Supplementary material. As depicted in Fig. 3B, the daily average AOC values at the mountainside and surface sites were (3.5 ± 1.3) × 10^7^ and (5.3 ± 2.1) × 10^7^ molecules cm^−3^ s^−1^, respectively. The AOC value on the mountainside was close to that observed in Shanghai (3.6 × 10^7^ molecules cm^−3^ s^−1^) (53) and a regional background site in Hong Kong (3.3 × 10^7^ molecules cm^−3^ s^−1^) (54). Nonetheless, both the mountainside and surface sites exhibited lower AOC values compared with more polluted cities, such as Xiamen (6.7 × 10^7^ molecules cm^−3^ s^−1^) (55) and Zhengzhou (7.4 × 10^7^ molecules cm^−3^ s^−1^) (36). Among the oxidants contributing to the AOC, OH radicals had the highest average concentration, accounting for 94.5 and 97.1% of the total AOC, followed by O_3_ (5.1 and 2.6%) and NO_3_ (0.4 and 0.2%) on the mountainside and at the surface, respectively.

The detailed O_3_ chemical production and destruction rates were quantified, as shown in Fig. 3C. The different reaction channels for O_3_ production and destruction are listed in the Supplementary material. The net O_3_ formation rate (NETO_3_) is defined as the difference between the O_3_ production rate (PO_3_) and the consumption rate (LO_3_). The daytime (06:00–19:00 LT) average NETO_3_ values were 5.6 ppbv h^−1^ on the mountainside and 7.4 ppbv h^−1^ at the surface, while the PO_3_ and LO_3_ at these two sites were 9.6 ppbv h^−1^, 3.9 ppbv  h^−1^ and 11.8 ppbv h^−1^, 4.4 ppbv h^−1^, respectively. The reaction of HO_2_ with NO was identified as the predominant pathway for PO_3_, contributing to 62.0% on the mountainside and 61.5% at the surface, which is similar to the results reported in the industrial city (Zibo) of the North China Plain (56). However, on high O_3_ pollution days, with elevated NO levels on the mountainside, the contribution of the HO_2_ + NO reaction dropped to 55.6%, compared with 61.4% at the surface. This marked reduction suggests a significant increase in the reaction rate of RO_2_ + NO under sufficient NO conditions, leading to enhanced O_3_ pollution on the mountainside. This trend is further illustrated by Fig. S3, which shows the changes in O_3_ formation and consumption pathways during O_3_ pollution periods, showing that the daytime average O_3_ formation rate increased by 25% to 12.0 ppbv h^−1^ on the mountainside, in contrast to a 15% increase to 13.6 ppbv h^−1^ at the surface. For LO_3_, major contributors included reactions of alkenes + O_3_, OH + NO_2_, O^1^D + H_2_O, and HO_2_ + O_3_. The contributions of different reaction channels at the two sites were distinct; specifically, the reaction of OH with NO_2_ dominated the destruction pathways at both sites, contributing 63.4% on the mountainside and 76.8% at the surface.

The relationship between O_3_ and its precursors at both sites was examined through sensitivity analysis, utilizing the relative increment reactivity (RIR) as a measure of how reductions in precursor concentrations influence O_3_ levels, as detailed in the Supplementary material. Figure 3D illustrates the RIRs of key species or groups affecting O_3_ formation, such as alkenes, isoprene, aromatics, OVOCs, NO*_x_*, and CO. The NO*_x_* value was positive on the mountainside and negative at the surface, signaling significant differences in O_3_ formation sensitivity and mechanisms between the two locations. The ratio of the RIR of NO*_x_* to the RIR of anthropogenic VOCs was used to identify the photochemical regimes (56). Values <0.5 indicate a VOC-limited regime, values above 2 suggest a NO*_x_*-limited regime, and values between 0.5 and 2 denote a transitional regime. In this study, the mountainside was in NO*_x_*-limited regime with the ratio of 2.8, whereas the surface site was in VOC-limited regime, with value of − 1.6. This implies that NO*_x_* plays a more important role in O_3_ formation within the PBL than at the surface. Similar conclusion can be found in previous vertical observational studies across Beijing, Hefei, Guangzhou, and Shanghai (13–17). Notably, the RIR value of isoprene was much higher than other compounds on the mountainside, indicating its substantial contribution to O_3_ formation. Studies have shown that isoprene emissions from mountainous and forested areas can significantly impact the oxidative capacity of the background atmosphere and elevate O_3_ concentrations in downwind cities (57, 58).

Given that daytime average isoprene concentration are about three times higher on the mountainside than at the ground, a replacement simulation experiment was conducted using the decayed ground-level isoprene concentration to quantify its significant contribution to O_3_ formation on the mountainside, as detailed in the Supplementary material. Referring to Fig. S4A, isoprene emitted from mountainside vegetation contributed ∼48.4% to the daytime O_3_ production on the mountainside. Additionally, as shown in Fig. S4B, by simulating the sensitivity of O_3_ formation to NO*_x_* under normal conditions and using decayed isoprene concentration, we found that isoprene emissions from the mountain enhance the sensitivity of BL O_3_ to NO*_x_*, with the average daytime RIR of NO*_x_* rising from 0.13 to 0.66. NO*_x_*, typically an anthropogenic emission from the surface, can spread to the mountainside as the BL expands. Due to the high sensitivity of mountainside O_3_ formation to NO*_x_*, this may result in a rapid increase in O_3_ levels. Previous studies have shown that vertical exchange of O_3_ from elevated altitudes can contribute to >20% of the variations in surface O_3_ (6, 8). Thus, exploring the distinct contributions of NO*_x_* to O_3_ formation on the mountainside is crucial for enhancing strategies to mitigate ground-level pollution.

### The crucial role of NO in O_3_ production on the mountainside

**Fig. 4.**
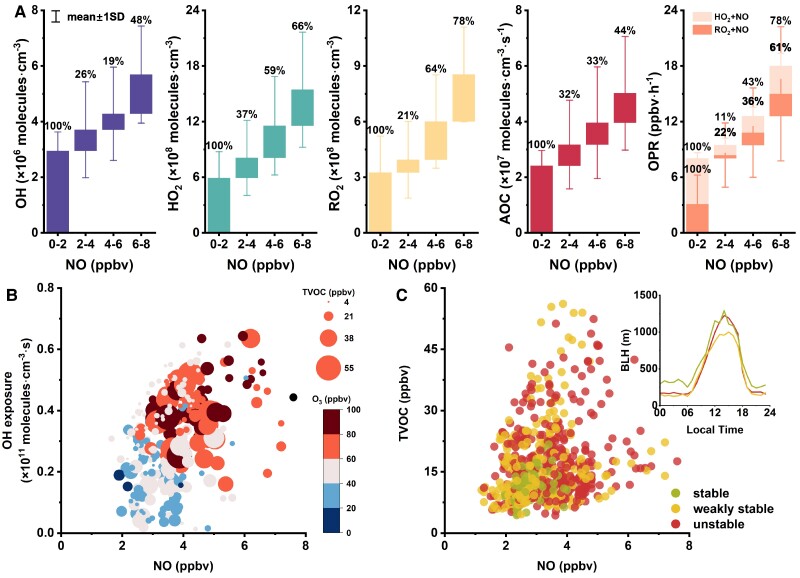
A) Mountainside concentrations of OH, HO_2_, and RO_2_ radicals; AOC and OPR with different NO concentrations. B) OH exposure as a function of NO concentration on the mountainside. The circles are colored according to the O_3_ concentration, and the circle sizes are scaled to the TVOC concentration. C) TVOC concentration as a function of NO concentration on the mountainside. The circles are colored according to atmospheric stability. Green, yellow, and red represent stable atmosphere, weakly stable atmosphere and unstable atmosphere, respectively.

Figure 4A illustrates the daytime variations in radical concentrations, AOC, and simulated ozone production rates (OPRs) under varying NO concentrations on the mountainside. An increase in NO concentration leads to a marked rise in radical concentrations, with RO_2_ radicals increasing more significantly than OH and HO_2_ radicals. This trend is mirrored in both AOC and OPR, demonstrating a consistent response to raising NO levels. Figure S5 further illustrates how escalating NO concentrations enhance the reactions between HO_2_ and RO_2_ with NO, accelerating the rate of radical cycling and, consequently, increasing O_3_ production. The OH exposure rate is indicative of air mass freshness, with higher rates suggesting more recent air mass transport (as discussed in the Potential sources of VOCs section). Figure 4B and C illustrate a positive correlation between elevated NO concentrations on the mountainside and air mass freshness. Moreover, the influx of freshly transported air masses with higher NO concentration to the mountainside, facilitated by both vertical and horizontal transport mechanisms, occurs under conditions of relatively low atmospheric stability.

The abundance of HO_2_ and RO_2_ radicals on the mountainside facilitates rapid engagement in radical cycling with incoming fresh air masses, leading to a transient yet significant increase in O_3_ levels. This phenomenon plays a critical role in influencing surface O_3_ concentrations via vertical air transport. Figure S6 depicts the fluctuations in O_3_ concentrations alongside potential sources at both the mountainside and the surface sites during an episode of O_3_ pollution marked by high NO*_x_* concentrations. The sustained elevation of O_3_ levels on the mountainside, coupled with the observed lag in peak values relative to other dates, confirms the extensive occurrence of photochemical reactions at this elevation.

**Fig. 5.**
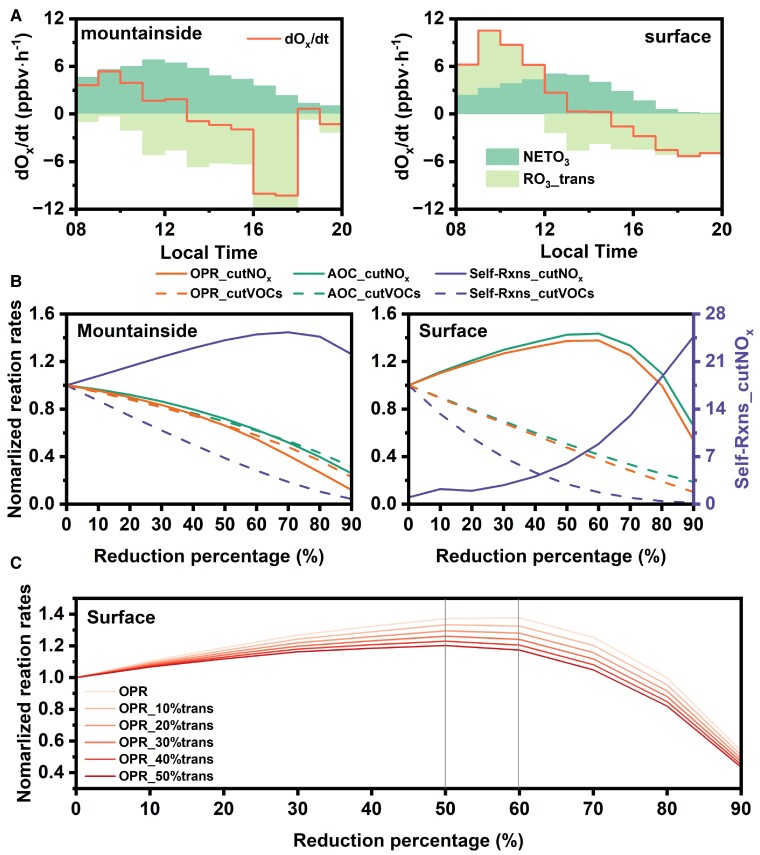
A) The sources of O_3_ at the two sites under high NO*_x_* conditions. The orange line represents the hourly variation rate of O_3_, the dark green bar represents the simulated net O_3_ production, and the light green bar represents the O_3_ transport. A positive O_3_ transport value indicates a net input, while a negative value represents a net output. B) Changes in OPR, AOC, and the total rate of Self-Rxns with reduction in NO*_x_* and VOCs at the mountainside and surface sites. Reaction rates are normalized to the corresponding values in the base run. The right axis in panel shows the normalized reaction rates for Self-Rxns upon NO*_x_* reduction at the surface. C) Changes in OPR with reductions in NO*_x_* at the surface, with colors ranging from light to dark representing an increasing percentage of the vertical contribution to the surface.

Moreover, the O_3_ levels on the mountainside diminishing through vertical exchange could contribute to an increase in surface O_3_ concentrations, as shown in Fig. 5A. This figure details the diurnal O_3_ variations at both sites under high NO*_x_* conditions on the mountainside, where “transported O_3_” is determined by the difference between the hourly O_3_ change rate and the in situ formation rate. Here, positive values indicate a net increase due to transport, whereas negative values suggest a net loss of O_3_. Notably, in the morning, the surface O_3_ concentration experienced a significant net gain from transport, likely stemming from vertical movement, as indicated by the negative transport rate on the mountainside.

## Discussion

With considerable efforts at emission mitigation (The State Council of China, 2013, 2018), China's annual average PM_2.5_ concentration significantly decreased, primarily due to reductions in SO_2_ emissions. However, O_3_ concentrations have shown a rising trend annually, prompting many studies to focus on emission control strategies for O_3_ precursors, NO*_x_* and VOCs. This study highlights the significant sensitivity of low-altitude O_3_ to NO levels, particularly in mountainous areas, underscoring the need for targeted attention.

Scenario simulations by proportionally reducing NO*_x_* and VOCs were conducted to compare the changes in OPR, AOC, and the total rate of self-reactions between peroxy radicals (Self-Rxns) on the mountainside and at ground level (as shown in Fig. 5B). The results show that reducing NO*_x_* significantly decreased OPR and AOC on the mountainside, confirming the high sensitivity of the mountainside O_3_ to NO*_x_*. At the surface, in line with the negative sensitivities to NO*_x_*, OPR and AOC increased with the reduction of NO*_x_* by 10–60%. However, further reducing NO*_x_* led to a rapid decrease in both OPR and AOC, indicating a shift in the O_3_ formation regime. It is striking that the rate of Self-Rxns would increase by >25-fold during NO*_x_* reduction at the surface. Although not significantly increased, the Self-Rxns rate would not be lowered by NO*_x_* reduction on the mountainside and remained stable at high levels. In contrast, reducing VOCs not only decreased OPR and AOC but also weakened the Self-Rxns at both sites. Similar results were simulated at two cities in eastern China, representing a NO*_x_*-rich and NO*_x_*-lean environment, respectively (13). The reduction of VOCs affected Self-Rxns because it limited the source of RO_2_ radicals, whereas reducing NO*_x_* increased RO_2_ production by elevating OH levels in the VOC-limited regime and decreased the conversion of peroxy radicals in the NO*_x_*-limited regime. Given that some Self-Rxns lead to secondary organic aerosol formation (59), controlling VOCs should be prioritized to achieve co-benefit for O_3_ and PM_2.5_ reduction.

The O_3_ levels on the mountainside exhibited marked sensitivity to NO. Given its status as a reservoir rich in HO_2_ and RO_2_ radicals, the mountainside was primed to rapidly produce O_3_ upon the arrival of fresh air masses containing elevated levels of NO*_x_*. This newly formed O_3_ was subsequently transported vertically to the surface. Due to the positive correlation between the OPR and NO*_x_* levels on the mountainside, reducing NO*_x_* emissions led to a rapid decrease in OPR on the mountainside. Conversely, at the surface, OPR initially increased and then decreased. Figure 5C shows the variation of the surface OPR with different NO*_x_* reduction ratios, considering the varying impacts of the vertical transport. It can be seen that, due to the differences in O_3_ formation sensitivity in the vertical direction, the peak of surface OPR is lower and occurs earlier (shifting from a 60% reduction to a 50% reduction) when the effect of vertical transport is more fully considered. This insight has implications for NO*_x_* reduction strategies at ground level, particularly in urban areas influenced by biogenic emissions from mountainous regions.

Therefore, it becomes crucial to implement more localized and refined emission reduction strategies that specifically address the variability in O_3_ sensitivity to NO across different atmospheric altitudes and its consequent impact on surface O_3_ levels. This approach will significantly enhance the effectiveness of air quality management efforts.

## Materials and methods

### Site description and instrumentation

The observation campaign was conducted at two stations, the mountainside station (36°22′52′′N, 117°10′39′′E, 520 m a.s.l.), and the surface station (36°12′40′′N, 117°02′16′′E, 160 m a.s.l.; Fig. S7), from 15 June to 31 August in 2022. As shown in Fig. S7, the mountainside site experiences diurnal variations, residing within the BL during the daytime and within the residual layer during the nighttime, rendering it subject to influences from both surface emissions and transported pollutants. The surface station is on the rooftop of a park building at the foot of the mountain, about 500 m from a major city road, with two highways 1 and 6 km away. There are no significant industrial emissions or residential buildings nearby. The mountainside station has an unobstructed view of the city below, with no residents, tourists, or obvious emission sources like restaurants around.

Online gas chromatographs equipped with a mass spectrometer and flame ionization detector (GC-MS/FID) system were utilized to continuously monitor ambient VOCs on the two sites. A total of 74 VOC species were quantified, including 29 alkanes, 10 alkenes, 1 ethyne, 16 aromatics, and 18 OVOCs (Table S1). Furthermore, criteria pollutants (O_3_, NO*_x_*, CO, and SO_2_) and meteorological parameters (*temperature*, relative humidity, wind direction, and wind speed) were also measured. More details of the measurements are found in the Supplementary material.

### In situ photochemistry modeling

Two campaign-tailored zero-dimensional chemical box models, one for the mountainside and another for the surface site, incorporating a subset of the MCM (version 3.3.1) (60, 61), were used to investigate radical production chemistry, utilizing the AtChem2 modeling toolkit (62). The observed mixing ratios of air pollutants, including O_3_, NO, NO_2_, CO, SO_2_, and measured VOCs were used to constrain the model. The temperature, relative humidity, and photolysis frequency of NO_2_ (as detailed in the Supplementary material) were also used as inputs to drive the model. The measurement data were averaged or linearly interpolated to a 1-h temporal resolution prior to inclusion in the models. The first 24 h of measurements were repeated for 3 days prior to the full campaign time series. The modeled data were then discarded before analysis to ensure that radical spin-up was achieved.

## Supplementary Material

pgae347_Supplementary_Data

## Data Availability

All data are included in the manuscript and supplementary material.
