# Supplementary material for: Vertical ozone formation mechanisms resulting from increased oxidation on the mountainside of Mount Tai, China
Source: PNAS Nexus. 2024 Aug 22;3(9):pgae347. doi: 10.1093/pnasnexus/pgae347 (PMC11376371; doi:10.1093/pnasnexus/pgae347)
Supplement: pgae347_Supplementary_Data [file pgae347_supplementary_data.docx]

**
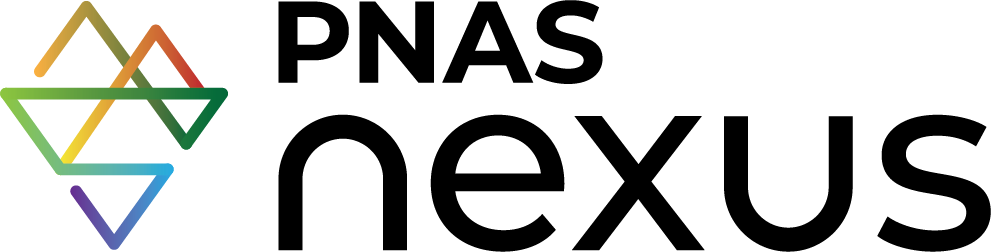
**

**Supplementary Information for**

Vertical ozone formation mechanisms resulting from increased oxidation on the mountainside of Mount Tai, China

Wanqi Wu^a^, Yanzhen Ge^b^, Yan Wang^c^, Jixin Su^c^, Xinfeng Wang^c^, Bin Zhou^a^, Jianmin Chen^a*^

^a^ Department of Environmental Science and Engineering, Shanghai Key Laboratory of Atmospheric Particle Pollution and Prevention (LAP^3^), Fudan University, Shanghai 200438, China.

^b^ Tai’an Ecological Environment Protection and Control Center, Tai’an Ecological Environment Bureau, Tai’an 271000, China.

^c^ School of Environmental Science and Engineering, Research Institute of Environment, Shandong University, Qingdao 266237, China.

*Jianmin Chen

**Email:** jmchen@fudan.edu.cn

**This PDF file includes:**

Supplementary text

Figures S1 to S7

Tables S1 to S3

SI References

**Supplementary Information Text**

**VOCs reactivity.** To determine the ozone formation capability of VOCs, Ozone Formation Potential (OFP) (1) and OH radical loss rate (*L*_OH_) (2)and were applied in this study. OFP is applied to calculate the maximum contribution to ozone formation*,* while *L*_OH_ could be used to calculate the chemical reactivity of VOCs. The calculative process is given in the following equations:

$${OFP}_{i}=\mathrm{VOC}_{i}\times{MIR}_{i}$$

$$L_{OHi}=\mathrm{VOC}_{i}\times K_{OHi}$$

where ${OFP}_{i}$ and $L_{OHi}$are the ozone formation and loss rate potential of the VOCs species i, respectively. $\mathrm{VOC}_{i}$represent the concentration of the VOC species i; and ${MIR}_{i}$is the maximum incremental reactivity of VOCs species i; $K_{OHi}$represents the rate constant of reactions with OH radicals of species i.

**OH exposure.** OH exposure represents OH radical concentration multiplied by the reaction time (Δt)(3). [OH] is the atmospheric OH concentration, and Δt represents the reaction time. [OH]Δt can be calculated from the ratios of specific VOCs with similar sources but with different reactivities. Here, we prefer to use the VOC pair consisting of m/p-xylene and ethylbenzene. This ratio can be determined as follows:

$$[OH] \Delta t= \frac{1}{k_{X}-k_{E}}\times\left[ \left. ln[X/E] \right|_{t=0}-ln[X/E] \right]$$

where the parameters $k_{X}$ and $k_{E}$ are the rate constants of m/p-xylene and ethylbenzene with OH radicals (cm^3^ molecule^−1^ s^−1^), respectively(2). $\left. [X/E] \right|_{t=0}$ is the initial mixing ratio of m/p-xylene to ethylbenzene. $[X/E]$ indicates the measured mixing ratio of m/p-xylene to ethylbenzene. As shown in Figure 2D, $\left. [X/E] \right|_{t=0}$ are 2.26 and 2.64 on the mountainside and the surface, respectively.

**Atmospheric oxidation capacity.** Atmospheric oxidation capacity (AOC) can be represented by the sum of the oxidation rates of molecules Y_i_ (VOCs and CO) with X_j_ (OH, O_3_, and NO_3_) (4):

$$AOC=\sum k_{Y_{ij}}\left[ Y_{i} \right]\left[ X_{j} \right]$$

where $k_{Y_{ij}}$ is the rate constant for the reaction of $Y_{i}$ (molecules cm^−3^) with $X_{j}$ (molecules cm^−3^); AOC, molecules cm^−3^ s^−1^. To evaluate the AOC, the concentrations of oxidants including O_3_, NO_3_, and OH radicals and primary gas pollutants including VOCs and CO are used(5).

**Ozone chemical production/destruction rates.** Production rate of O_3_ (P(O_3_)) was determined as the sum of the rates of HO_2_+NO and RO_2_+NO. The O_3_ destruction rate (L(O_3_)) was determined to be the sum of the rates of O_3_ photolysis (represented by O^1^D loss to H_2_O), reactions with radicals (O_3_+OH/HO_2_), reactions with alkenes (O_3_+alkenes) and NO_2_+OH. The net O_3_ formation rate (NET(O_3_)) was calculated as the difference between P (O_3_) and L (O_3_):

$$P\left( O_{3} \right)=k_{1}\left[ HO_{2} \right]\left[ NO \right]+\sum(k_{2i}\left[ RO_{2} \right]_{i}\left[ NO \right])$$

$$L\left( O_{3} \right)=k_{3}\left[ O^{1}D \right]\left[ H_{2}O \right]+k_{4}\left[ O_{3} \right]\left[ OH \right]+k_{5}\left[ O_{3} \right]\left[ HO_{2} \right]+\sum(k_{6i}\left[ O_{3} \right]\left[ \mathrm{alkenes} \right]_{i})+k_{7}\left[ NO_{2} \right]\left[ OH \right]$$

$$NET\left( O_{3} \right)=P\left( O_{3} \right)-L\left( O_{3} \right)$$

where $k_{i}$ is the reaction rate coefficient of different reactions derived from the MCM website (<http://mcm.york.ac.uk/>).

The variations in O_3_ concentration can reflect the combined effect of photochemical and physical transport processes on O_3_ abundance. This variation can be expressed as followed:

$$\frac{d\left( O_{3} \right)}{d\left( t \right)}=NET\left( O_{3} \right)+{R\left( O_{3} \right)}_{trans}$$

R(O_3_)_trans_ was identified as the rate of physical transport of O_3_, which was determined by subtracting P(O_3_) from d(O_3_)/d(t). A positive value of R(O_3_)_trans_ indicated that O_3_ abundance at a specific location could be partly contributed by air masses transport(6).

**Relative increment reactivity (RIR).** RIR value is used to diagnose the sensitivity of O_3_ formation to specific precursors. It is defined as the percent change in the O_3_ production rate divided by the percent change in precursor concentrations, as shown in the following equations:

$$RIR(X)=\frac{\left[ P_{O_{3}}\left( X \right)-P_{O_{3}}\left( X-\Delta X \right) \right]/P_{O_{3}}\left( X \right)}{\Delta X/X}$$

where $\Delta X/X$ is the reduction ratio of specific precursor X (10% was chosen in this study). A positive RIR implies that more precursors lead to increasing O_3_ formation, whereas when a negative RIR means the growth of precursors inhibits formation. Greater RIR suggests greater sensitivity to specific precursors.

**Replacement simulation experiment.** Because the mountain is a typical source of biogenic VOCs during the summer, we found higher isoprene concentrations at the mountainside level, which is different from the atmospheric composition above most cities. Therefore, the conclusion that "mountainside O_3_ production is highly sensitive to NO_x_ " cannot be directly compared or discussed with existing results based on the formaldehyde-to-NO_2_ ratio, which suggest that " O_3_ production within the urban boundary layer is in the transitional or NO_x_-limited regime." We conducted replacement simulation experiments to quantify its significant contribution to O_3_ formation on the mountainside. In this process, the isoprene concentration at the mountainside model input parameters is replaced with the concentration decayed from ground-level isoprene, with the decay coefficient referenced from literature on isoprene vertical profiles(7).

$$Isoprene_{mountainside\_decay}=Isoprene_{surface}*0.48$$

**Measurement.** Online gas chromatographs equipped with a [mass spectrometer](https://www.sciencedirect.com/topics/earth-and-planetary-sciences/mass-spectrometer) and [flame ionization](https://www.sciencedirect.com/topics/earth-and-planetary-sciences/flame-ionization) detector (GC–MS/FID) System ZF-PKUVOC1007 (Pengyu Changya Inc., Beijing, China) and TH-300B (Tianhong Inc., Wuhan, China) were utilized to continuously monitor ambient VOCs on the mountainside and surface, respectively. Light (C_2_-C_5_) VOCs were quantified by the FID, and C_5_-C_12_ hydrocarbons, and oxygenated VOCs (OVOCs) were analyzed using the MS detector.

Hourly gaseous pollutants (O_3_, NO-NO_2_-NO_x_, CO, SO_2_) were detected by the gas analyzer (49i/42i/48i/43i, Thermo Fisher, USA). The temperature (T,°C), relative humidity (RH, %), wind speed (WS, m s^−1^) and wind direction (WD, °) were measured by the [automatic weather station](https://www.sciencedirect.com/topics/earth-and-planetary-sciences/automatic-weather-stations).

The photolysis frequencies of NO_2_ (J4) were calculated using the tropospheric ultraviolet and visible radiation model (TUV) and O_3_ column concentration data from ozone monitoring instrument (OMI, <https://www.temis.nl/index.php>) satellite observations.


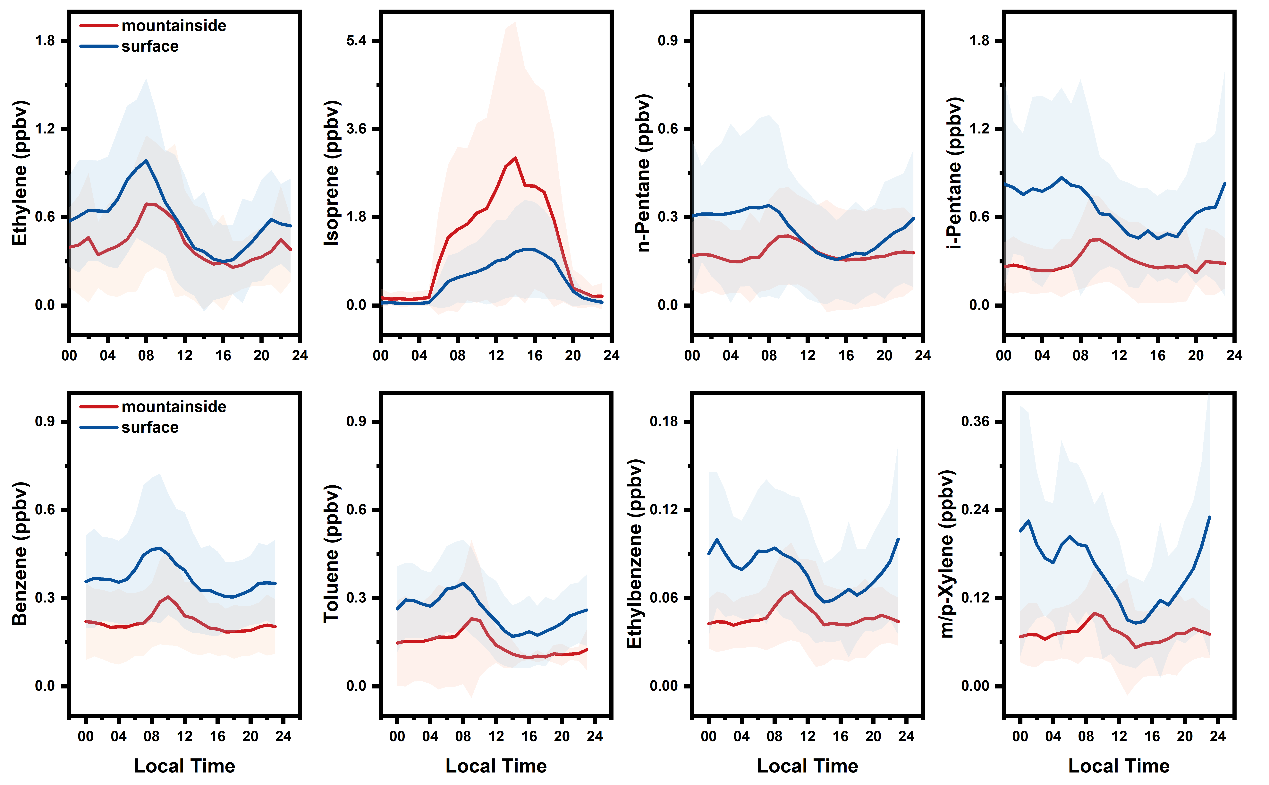


**Fig.** **S1.** Diurnal patterns of selected VOC species. Lines represent the average values, and shaded areas represent the standard deviations.


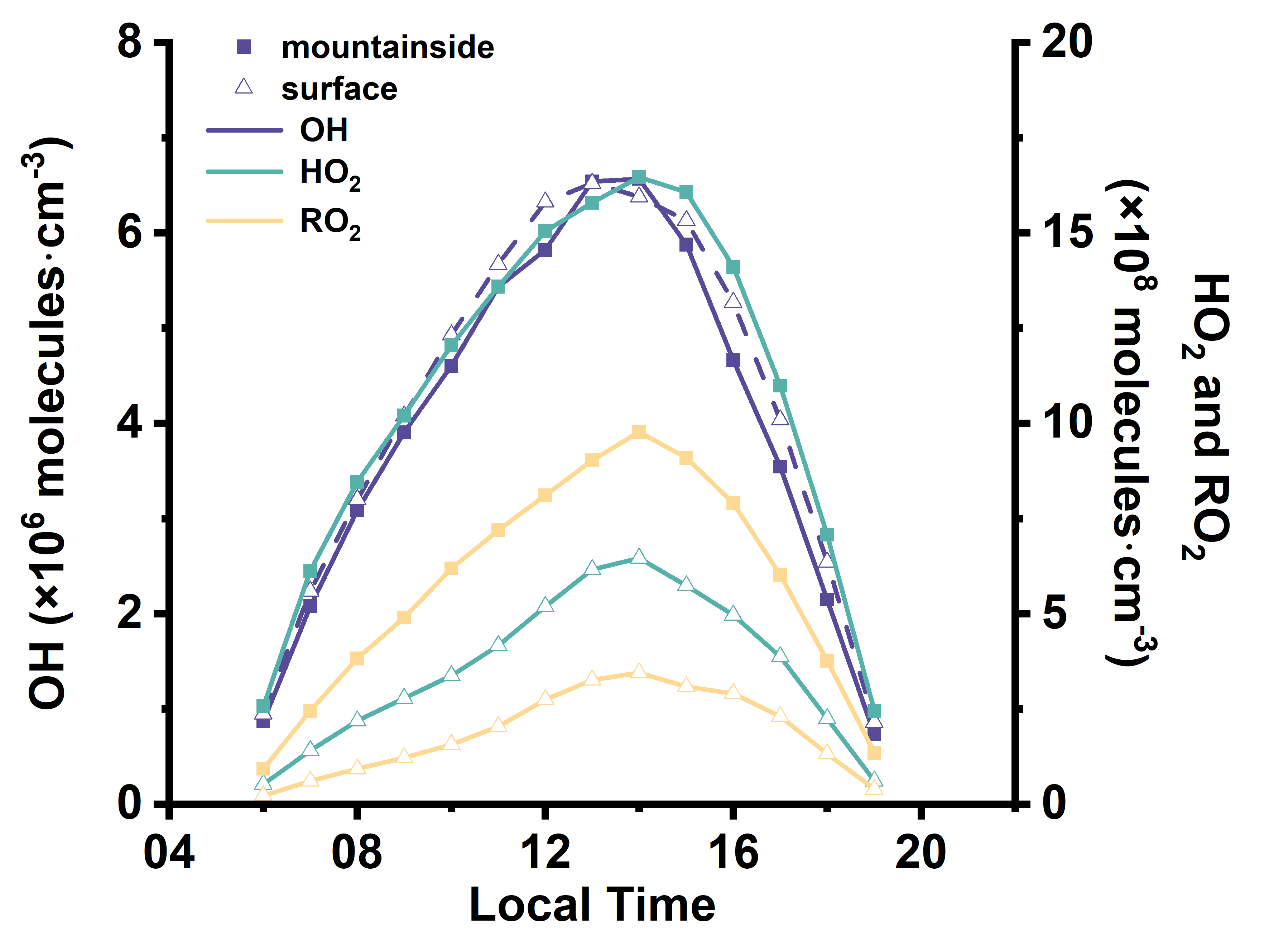


**Fig. S2.** Daytime variations in the concentrations of OH, HO_2_, and RO_2_ radicals on high O_3_ pollution days.


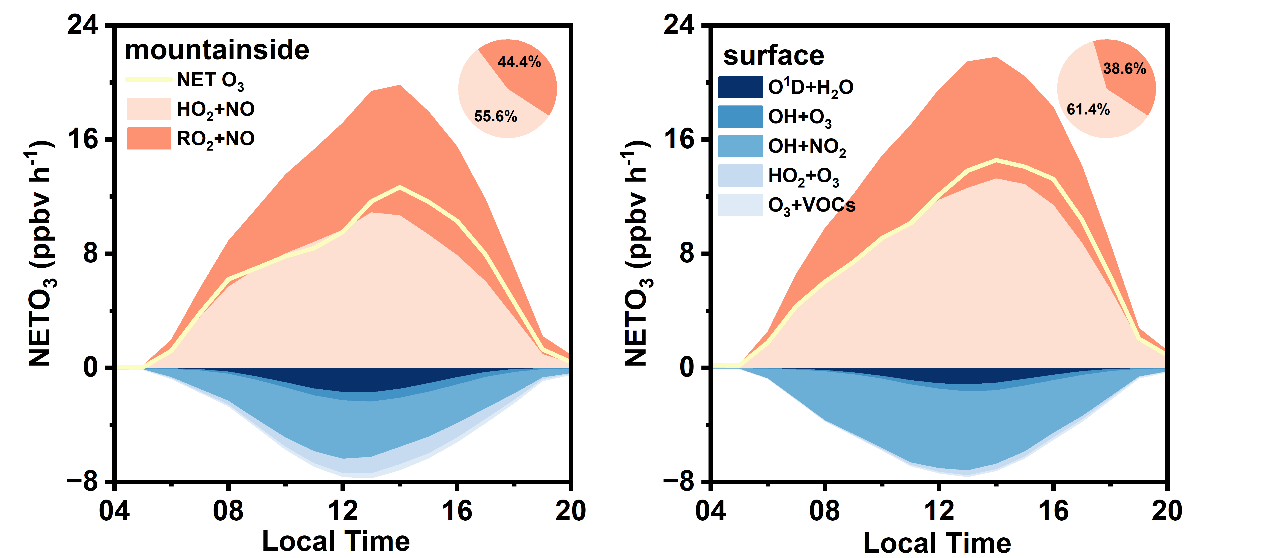


**Fig. S3.** The hourly variation in O_3_ production and destruction rates on the mountainside and at the surface on high O_3_ pollution days. Pie charts represent the portions of different reaction channels. The yellow lines represent the net O_3_ generation rate.


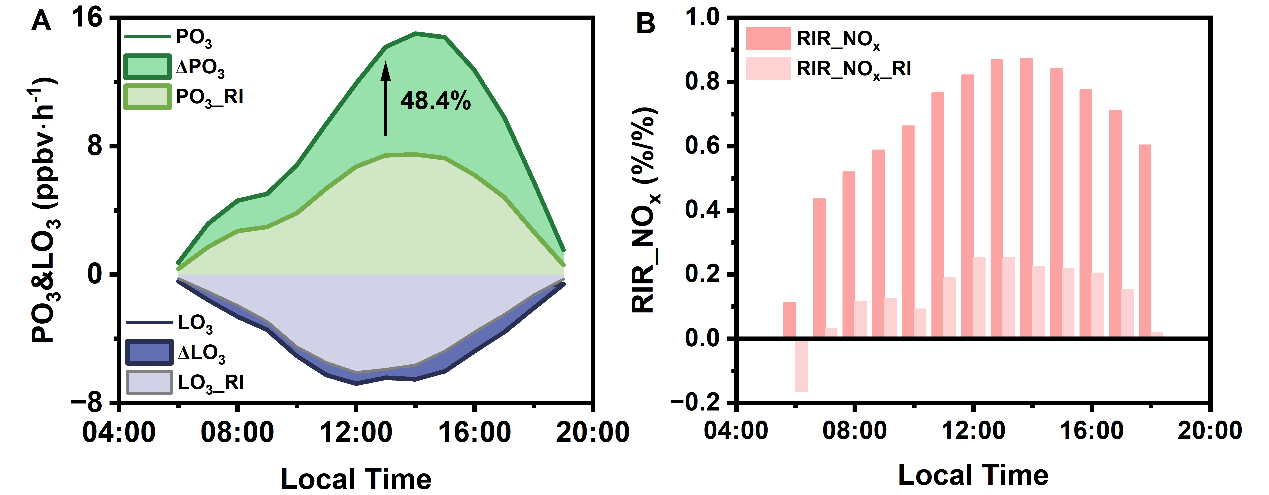


**Fig. S4.** (A) The simulated diurnal variation of the O_3_ production (PO_3_) and loss rates (LO_3_) on the mountainside under normal conditions and with reduced isoprene (RI) concentrations using ground-level isoprene decay during the daytime. Here, ΔPO_3_ andΔLO_3_ represent the contributions of isoprene emitted from mountainside vegetation to O_3_ production and consumption, respectively.(B) Relative increase in reactivity (RIR) of the NO_x_ under normal and RI conditions during high-O_3_ daytime (06:00–18:00 LT).


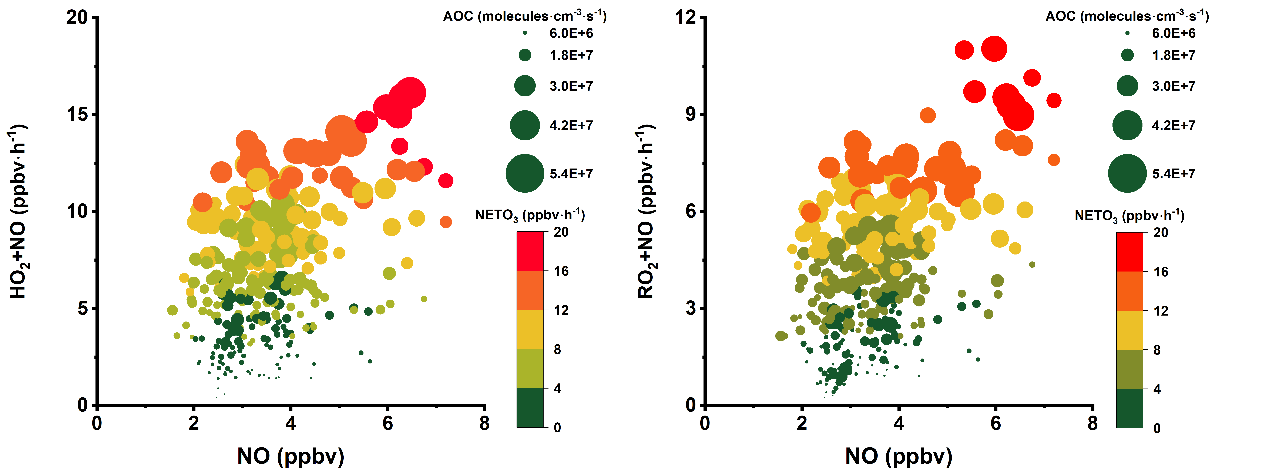


**Fig. S5.** The rate of HO_2_ with NO and RO_2_ with NO as a function of NO concentration on the mountainside. The circles are colored according to calculated in-situ net O_3_ production, and circle sizes are scaled to AOC.


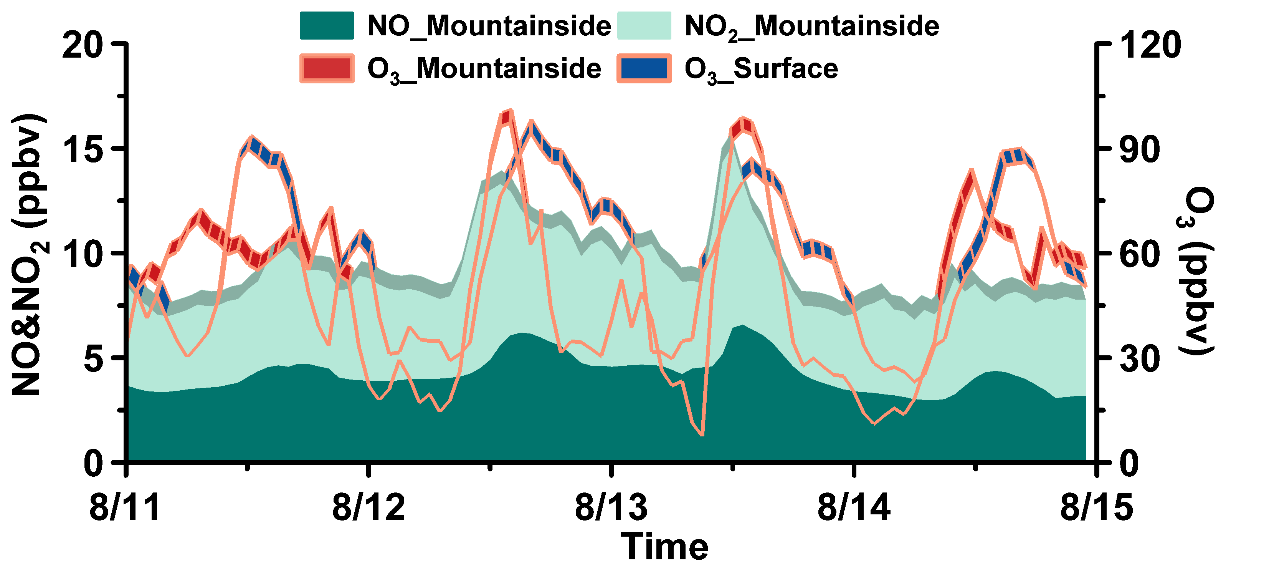


**Fig. S6.** Diurnal variations in O_3_ concentrations on the mountainside and at the surface during a NO_x_ pollution episode from August 11 to August 14.


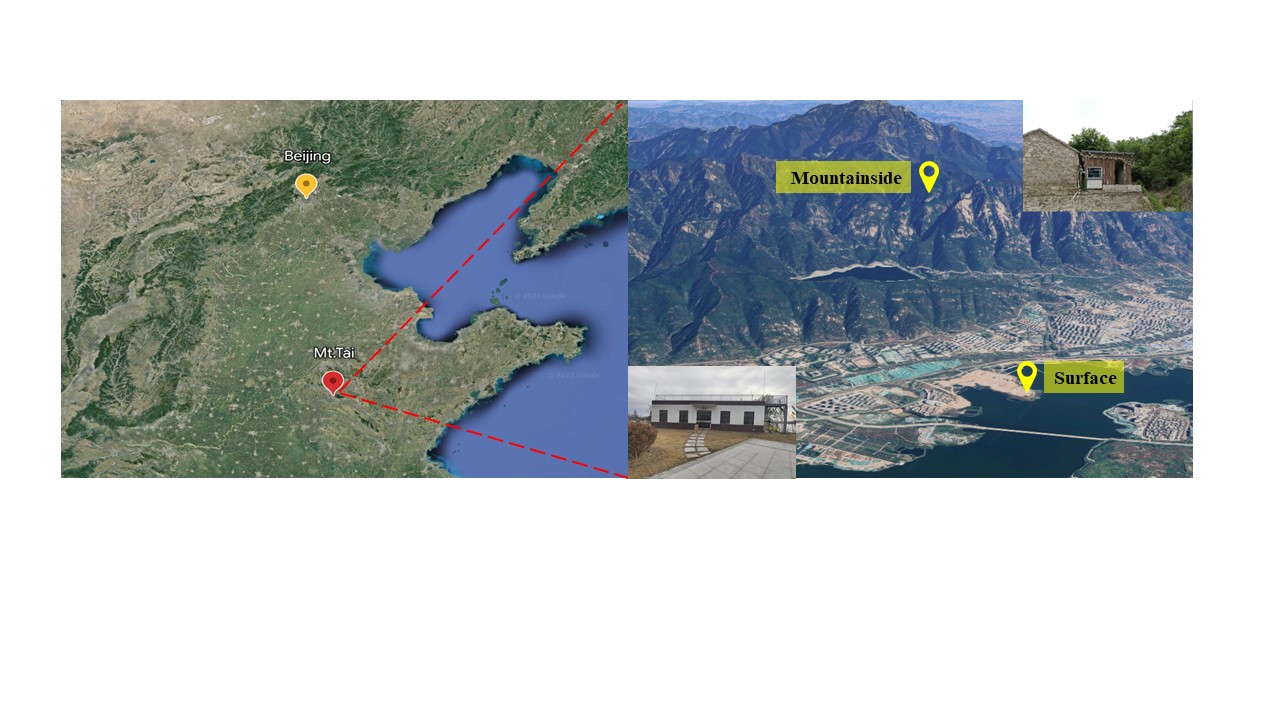


**Fig. S7.** Location of the sampling site.

**Table S1.** Concentrations of VOCs at the two sites.

| Group | Species | Mountainside (ppbv) | | Surface (ppbv) | |
| --- | --- | --- | --- | --- | --- |
|  |  | mean | SD | mean | SD |
| Alkanes | Ethane | 1.42 | 0.68 | 1.47 | 0.61 |
|  | Propane | 1.49 | 1.22 | 1.86 | 1.32 |
|  | i-Butane | 0.27 | 0.19 | 0.41 | 0.32 |
|  | n-Butane | 0.50 | 0.37 | 0.73 | 0.57 |
|  | i-Pentane | 0.30 | 0.23 | 0.66 | 0.51 |
|  | n-Pentane | 0.18 | 0.15 | 0.25 | 0.23 |
|  | 2,2-Dimethylbutane | 0.02 | 0.01 | 0.03 | 0.03 |
|  | 2,3-Dimethylbutane | 0.02 | 0.01 | 0.04 | 0.02 |
|  | Cyclopentane | 0.17 | 0.10 | 0.30 | 0.17 |
|  | 2-Methylpentane | 0.07 | 0.06 | 0.12 | 0.08 |
|  | 3-Methylpentane | 0.04 | 0.03 | 0.06 | 0.04 |
|  | n-Hexane | 0.10 | 0.09 | 0.10 | 0.07 |
|  | Methylcyclopentane | 0.02 | 0.01 | 0.04 | 0.03 |
|  | 2,4-Dimethylpentane | 0.01 | 0.00 | 0.02 | 0.01 |
|  | Cyclohexane | 0.04 | 0.02 | 0.04 | 0.02 |
|  | 2-Methylhexane | 0.02 | 0.01 | 0.03 | 0.02 |
|  | 2,3-Dimethylpentane | 0.01 | 0.01 | 0.02 | 0.01 |
|  | 3-Methylhexane | 0.02 | 0.01 | 0.03 | 0.02 |
|  | 2,2,4-Trimethylpentane | 0.02 | 0.01 | 0.02 | 0.01 |
|  | n-Heptane | 0.03 | 0.02 | 0.04 | 0.02 |
|  | Methylcyclohexane | 0.01 | 0.00 | 0.02 | 0.01 |
|  | 2,3,4-Trimethylpentane | 0.01 | 0.00 | 0.01 | 0.01 |
|  | 2-Methylheptane | 0.01 | 0.00 | 0.02 | 0.01 |
|  | 3-Methylheptane | 0.02 | 0.01 | 0.04 | 0.02 |
|  | n-Octane | 0.01 | 0.00 | 0.02 | 0.01 |
|  | Nonane | 0.01 | 0.00 | 0.02 | 0.01 |
|  | n-Decane | 0.01 | 0.00 | 0.01 | 0.01 |
|  | n-Undecane | 0.01 | 0.00 | 0.01 | 0.01 |
|  | n-Dodecane | 0.01 | 0.00 | 0.01 | 0.02 |
| Alkenes | Ethene | 0.42 | 0.35 | 0.58 | 0.42 |
|  | Propene | 0.17 | 0.12 | 0.40 | 0.22 |
|  | 1-Butene | 0.04 | 0.04 | 0.06 | 0.04 |
|  | trans-2-butene | 0.00 | 0.01 | 0.02 | 0.02 |
|  | cis-2-butene | 0.00 | 0.00 | 0.02 | 0.01 |
|  | 1-Pentene | 0.00 | 0.00 | 0.01 | 0.01 |
|  | Isoprene | 1.20 | 1.79 | 0.52 | 0.71 |
|  | trans-2-Pentene | 0.00 | 0.01 | 0.01 | 0.01 |
|  | cis-2-Pentene | 0.00 | 0.00 | 0.01 | 0.01 |
|  | 1-Hexene | 0.00 | 0.00 | 0.01 | 0.01 |
| Acetylene | Acetylene | 0.68 | 0.51 | 0.59 | 0.52 |
| Aromatics | Benzene | 0.22 | 0.12 | 0.37 | 0.17 |
|  | Toluene | 0.14 | 0.21 | 0.25 | 0.14 |
|  | m/p-Xylene | 0.07 | 0.05 | 0.15 | 0.11 |
|  | Styrene | 0.01 | 0.00 | 0.03 | 0.02 |
|  | o-Xylene | 0.05 | 0.03 | 0.08 | 0.05 |
|  | Isopropylbenzene | 0.01 | 0.00 | 0.02 | 0.01 |
|  | n-Propylbenzene | 0.01 | 0.00 | 0.01 | 0.01 |
|  | m-Ethyltoluene | 0.01 | 0.00 | 0.02 | 0.01 |
|  | Ethylbenzene | 0.05 | 0.02 | 0.08 | 0.04 |
|  | p-Ethyltoluene | 0.01 | 0.00 | 0.02 | 0.01 |
|  | 1,3,5-Trimethylbenzene | 0.01 | 0.00 | 0.02 | 0.01 |
|  | o-Ethyltoluene | 0.01 | 0.00 | 0.01 | 0.01 |
|  | 1,2,4-Trimethylbenzene | 0.02 | 0.01 | 0.03 | 0.02 |
|  | 1,2,3-Trimethylbenzene | 0.01 | 0.00 | 0.01 | 0.01 |
|  | m-Diethylbenzene | 0.06 | 0.01 | 0.09 | 0.03 |
|  | p-Diethylbenzene | 0.01 | 0.00 | 0.01 | 0.01 |
| OVOCs | Acetaldehyde | 1.60 | 0.92 | 2.56 | 0.95 |
|  | Acrolein | 0.09 | 0.08 | 0.10 | 0.05 |
|  | Propionaldehyde | 0.33 | 0.40 | 0.48 | 0.19 |
|  | Acetone | 4.93 | 4.61 | 3.65 | 1.17 |
|  | 2-Propanol | 0.13 | 0.12 | 0.12 | 0.05 |
|  | MTBE | 0.14 | 0.14 | 0.17 | 0.14 |
|  | Vinyl acetate | 0.16 | 0.12 | 0.18 | 0.07 |
|  | Methacrolein | 0.34 | 0.23 | 0.22 | 0.13 |
|  | Butyraldehyde | 0.13 | 0.10 | 0.12 | 0.05 |
|  | Ethyl acetate | 0.15 | 0.35 | 0.17 | 0.09 |
|  | 2-Butanone | 0.46 | 0.36 | 0.47 | 0.20 |
|  | Tetrahydrofuran | 0.02 | 0.02 | 0.03 | 0.02 |
|  | Methyl methacylate | 0.02 | 0.55 | 0.03 | 0.01 |
|  | Valeraldehyde | 0.08 | 1.16 | 0.10 | 0.05 |
|  | 1,4-Dioxane | 0.02 | 0.52 | 0.05 | 0.01 |
|  | 4-Methyl-2-pentanone | 0.03 | 0.03 | 0.03 | 0.02 |
|  | 2-Hexanone | 0.01 | 0.01 | 0.02 | 0.02 |
|  | n-Hexaldehyde | 0.10 | 0.13 | 0.09 | 0.05 |

**Table S2.** Concentrations of VOC groups at the two sites.

| Group | Mountainside (ppbv) | | Surface (ppbv) | |
| --- | --- | --- | --- | --- |
|  | mean | SD | mean | SD |
| Alkanes | 4.82 | 2.70 | 6.44 | 3.26 |
| Alkenes | 0.64 | 0.45 | 1.11 | 0.60 |
| Acetylene | 0.68 | 0.51 | 0.59 | 0.52 |
| Aromatics | 0.68 | 0.35 | 1.20 | 0.49 |
| Isoprene | 1.20 | 1.79 | 0.52 | 0.70 |
| OVOCs | 8.75 | 5.83 | 8.59 | 2.52 |
| Total | 16.77 | 8.31 | 18.45 | 6.11 |

**Table S3.** OFP and OH radical loss rate of VOC groups at the two sites.

| Group | Mountainside | | Surface | |
| --- | --- | --- | --- | --- |
|  | OFP (μg/m^3^) | L_OH_ (s⁻¹) | OFP(μg/m^3^) | L_OH_ (s⁻¹) |
| Alkanes | 10.12±5.59 | 0.23±0.13 | 15.90±8.14 | 0.36±0.18 |
| Alkenes | 9.67±6.23 | 0.24±0.15 | 19.14±9.37 | 0.53±0.25 |
| Acetylene | 0.76±0.56 | 0.03±0.02 | 0.66±0.57 | 0.03±0.02 |
| Aromatics | 13.42±5.94 | 0.28±0.14 | 23.42±10.23 | 0.52±0.26 |
| Isoprene | 38.65±57.78 | 2.95±4.41 | 16.86±22.82 | 1.29±1.74 |
| OVOCs | 48.53±29.58 | 2.70±1.88 | 71.59±19.77 | 3.02±0.83 |
| Total | 119.13±82.91 | 6.32±5.75 | 147.52±46.75 | 5.72±1.77 |

**SI References**

1. W. P. L. Carter, Development of the SAPRC-07 chemical mechanism. *Atmospheric Environment* **44**, 5324–5335 (2010).

2. R. Atkinson, J. Arey, Atmospheric Degradation of Volatile Organic Compounds. *Chem. Rev.* **103**, 4605–4638 (2003).

3. C. Li, *et al.*, Divergent summertime surface O3 pollution formation mechanisms in two typical Chinese cities in the Beijing-Tianjin-Hebei region and Fenwei Plain. *Science of The Total Environment* **870**, 161868 (2023).

4. A. Geyer, *et al.*, Chemistry and oxidation capacity of the nitrate radical in the continental boundary layer near Berlin. *J. Geophys. Res.* **106**, 8013–8025 (2001).

5. L. Kong, *et al.*, Atmospheric oxidation capacity and secondary pollutant formation potentials based on photochemical loss of VOCs in a megacity of the Sichuan Basin, China. *Science of The Total Environment* **901**, 166259 (2023).

6. W. Ma, *et al.*, Influence of photochemical loss of volatile organic compounds on understanding ozone formation mechanism. *Atmos. Chem. Phys.* **22**, 4841–4851 (2022).

7. Vertical distribution of isoprene in the lower boundary layer of the rural and urban southern United States. *Journal of Geophysical Research: Atmospheres* **99**, 16989–16999 (1994).
